# First Case of Autoimmune Hemolytic Anemia Associated With COVID-19 Infection in Sri Lanka: A Case Report

**DOI:** 10.7759/cureus.19118

**Published:** 2021-10-29

**Authors:** Vaishnavi Arunpriyandan, Somasuriyam Kumanan, Mayurathan Pakkiyaretnam

**Affiliations:** 1 Medicine, University Medical Unit, Faculty of Health-Care Sciences, Teaching Hospital Batticaloa, Batticaloa, LKA

**Keywords:** autoimmune hemolytic anemia, steroids, immune-mediated hemolysis, warm autoimmune hemolytic anemia, covid-19

## Abstract

Autoimmune hemolytic anemia (AIHA) is a condition characterized by the increased destruction of red blood cells (RBCs) mediated by anti-erythrocyte autoantibodies with or without complement activation. Its clinical presentation is heterogeneous, ranging from asymptomatic to severe forms with fatal outcomes, and it can be either idiopathic or secondary to a coexisting disorder. In this report, we present a case of a patient who suffered from her first episode of acute and severe AIHA during Severe Acute Respiratory Syndrome Coronavirus - 2 (SARS-CoV-2) and responded well with the treatment.

## Introduction

SARS-CoV-2 (also known as SARS COVID-19) is an evolving agent that is responsible for the ongoing pandemic for the last two years. Ever since, fascinating yet life-threatening complications have been added up to the medical literature from across the world. COVID-19 infection primarily causes respiratory symptoms and so frequently encountered complications are, pneumonia, acute respiratory distress syndrome, and respiratory failure [1]. Although the primary pathophysiology causing severe disease in COVID‐19 remains unclear, prevailing evidence supports an event of acute cytokine storm being associated with disease severity and negative outcome [2]. However, the range of complications is wider and includes amongst other several auto‐immune disorders such as autoimmune thrombocytopenia, Guillain-Barré syndrome, and anti-phospholipid syndrome [2,3]. Here we report a case of a young woman with autoimmune hemolytic anemia (AIHA) during COVID-19 infection for the first time in Sri Lanka.

## Case presentation

A 23-year-old previously healthy Sri Lankan female presented with fever and cough for three days to our Emergency Treatment Unit. Her rapid antigen test and polymerase chain reaction (PCR) for COVID-19 were positive and she was managed as moderately severe covid infection at ICU with noninvasive ventilation and high flow nasal oxygen (HFNO); however, invasive ventilation was not needed. On day 20, her COVID-19 exit PCR was negative and she was transferred to the general medical ward for the continuation of treatment.

While recovering from moderate to severe covid infection, she developed excessive fatigue and shortness of breathing. She had severe pallor and tachycardia on examination. She was oxygen-independent and there were no signs of hypoxia in her arterial blood gas. Her ECG showed normal sinus rhythm and the troponin I was negative. Initial blood investigations revealed an acute drop of hemoglobin to 5.1 g/dl from her baseline of 11 g/dl. White blood cell count and the platelet counts were within normal limits. There was no other evidence of any cardiac or respiratory disease. Transthoracic echocardiogram showed normal left ventricular function with no evidence of pulmonary embolism and the D-dimers were within normal limits. Her high-resolution computer tomography of the chest revealed only resolving consolidation. Her thyroid status was also found to be euthyroid.

On further history taking, there was no evidence of any internal or external bleeding. There is no family or past history of hematological malignancy or connective tissue disorders. In summary, she was a relatively healthy female with no risk factors for AIHA until she developed COVID-19.

**Figure 1:**
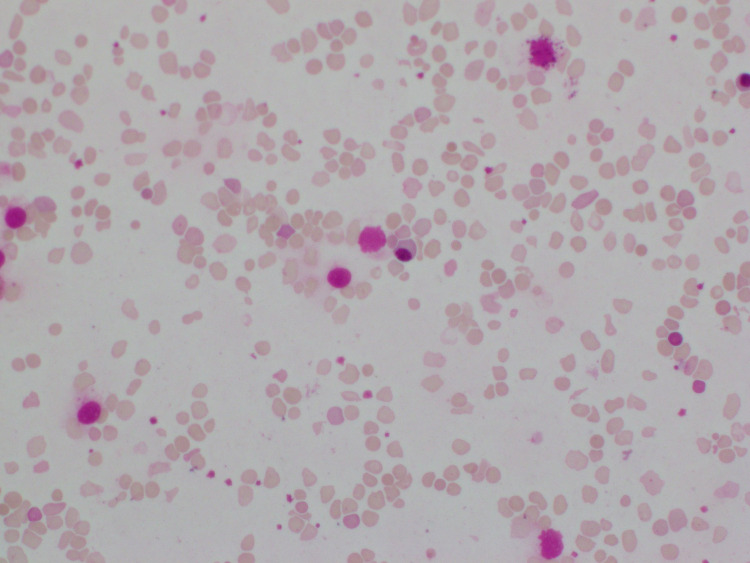
Blood picture

Her initial blood tests showed evidence of hemolysis with high reticulocyte count, lactate dehydrogenase (LDH), and indirect hyperbilirubinemia. A blood smear confirmed the presence of severe hemolytic anemia. The direct antiglobulin test was positive. Her Coombs profile confirmed warm-type autoimmune hemolytic anemia with complement 3b. Summary of the investigation results and the blood picture are shown below in Table 1 and Figure 1, respectively.

**Table 1:** Investigations according to the timeline MCV: mean corpuscular volume; MCH: mean corpuscular hemoglobin; MCHC: mean corpuscular hemoglobin concentration; LDH: lactate dehydrogenase; AST: aspartate aminotransferase; ALT: alanine aminotransferase; INR: international normalized ratio; CECT: contrast-enhanced computed tomography; ANA: anti-nuclear antibodies

Laboratory investigation	On admission	DAY 20	On discharge	Normal values
Hemoglobin	11.7g/dL	5.0 g/dL	10.8g/dL	11-15g/dL
MCV	87 fL	92.8 fL	88 fL	80-100fL
MCH	28.6 pg	28 pg	30 pg	27-34pg
MCHC	32.9 g/dL	32 g/dL	33g/dL	32-36g/dL
WBC	34 300/µL (79% Neutrophils)	23 000/µL ( 88% neutrophils)	11000/µL (normal differential count)	4000-11000/µL
Platelet	172000/ µL	152000/ µL	160000/ µL	150000-450000/ µL
Reticulocyte	-	8.24%		0.3-3%
LDH	-	979 U/L	100 U/L	81-234 U/L
Blood picture	-	Evidence of Severe Hemolysis	-	-
AST	25 U/L	473 U/l		15-37 U/L
ALT	50 U/L	102 U/l		12-78 U/L
Indirect bilirubin	-	20.2 µmol/L	7 µmol/L	3-14 µmol/L
INR	1.1	0.99	1	0.8-1.1
2D Echocardiogram	-	Good Left ventricular and right ventricular function	-	-
CECT -chest,abdomen,pelvis	-	Normal	-	-
ANA	-	11 AU/ml	-	0-40 AU/ml
Rheumatoid factor	-	Negative	-	-
Hepatitis B surface antigen	-	Negative	-	-
Mycoplasma antibody	-	Negative	-	-
HIV screening	-	Negative	-	-

She was managed with red cell transfusions and corticosteroid therapy. We considered rituximab as a second-line response. However, there was a good response for corticosteroid therapy (oral prednisolone 1 mg/kg). The hemoglobin went up to 10 g/dl with five days of treatment. We tapered off the prednisolone over a period of three months. On subsequent follow-up, she had stable hemoglobin.

## Discussion

Autoimmune hemolytic anemia is a rare autoimmune disorder that could occur in association with several autoimmune, malignant, or infectious diseases such as human immunodeficiency virus, mycoplasma, hepatitis B, cytomegalovirus, and parvovirus [3,4]. Association with novel coronavirus is rare [5,6].

There are few pieces of evidence in recent literature for AIHA occurring during SARS-CoV-2 infection. According to Quinn and Murakhovskaya, around 31 cases have been reported as AIHA occurring during the acute SARS-CoV-2 infection. The majority of them were of warm AIHA (n=16) and the rest were found to be cold agglutinin syndrome (n=15) [6]. As evidenced by the Coombs test, IgG and complement became positive in eight out of 14 patients, IgA in one patient, and IgG alone in five patients [6].

The average age of presentation in adults was around 63 years [6] in literature whereas our patient was a young female, and the majority presented within the first two weeks of the onset of the symptoms of SARS-CoV-2 infection [6]. To the best of our knowledge, this is the third report showing a delayed onset of AIHA (twenty-fifth day from symptom onset). Moreover, in most relevant cases reported so far, the patients suffered from malignant or lymphoproliferative disorders such as chronic lymphocytic leukemia, monoclonal gammopathy of undetermined significance, and one patient had a prior history of congenital thrombocytopenia [2,7]. In contrast, our patient did not have any predisposing conditions and probable risk factors. Overall, the occurrence of autoimmune hemolytic anemia in SARS-CoV-2 infection is uncommon, and among them, late-onset disease, especially in a healthy individual, is a very rare phenomenon.

The mainstay of therapy includes steroids and blood transfusion. Intravenous immunoglobulin was tried in one patient with a suboptimal response, which eventually responded to steroids [2,8]. Rituximab is considered the second-line agent. Plasma exchange has been tried in one patient. All reported patients convalesced from both SARS-CoV-2 and hemolysis so far [8]. Similarly, our patient also showed a good response to oral prednisolone 1 mg/kg and had stable hemoglobin on her subsequent follow-ups.

## Conclusions

In summary, although rare, AIHA coexisting with COVID-19 is a recently expressed phenomenon and it should be suspected in the context of unexplained or persistent anemia in patients with a current or recent history of COVID-19. In our experience, there is a good response to steroids and AIHA might be considered even when the COVID-19 acute phase is over. The rapid emergence and spread of SARS-CoV-2 have highlighted the importance of being prepared for any future event, being able to identify novel complications early, and addressing the risk factors that contribute to their emergence.

## References

[1] Zhou F, Yu T, Du R (2020). Clinical course and risk factors for mortality of adult inpatients with COVID-19 in Wuhan, China: a retrospective cohort study. Lancet.

[2] Lazarian G, Quinquenel A, Bellal M (2020). Autoimmune haemolytic anaemia associated with COVID-19 infection. Br J Haematol.

[3] Huda Z, Jahangir A, Sahra S, Rafay Khan Niazi M, Anwar S, Glaser A, Jahangir A (2021). A case of COVID-19-associated autoimmune hemolytic anemia with hyperferritinemia in an immunocompetent host. Cureus.

[4] Liebman HA, Weitz IC (2017). Autoimmune hemolytic anemia. Med Clin North Am.

[5] Jawed M, Hart E, Saeed M (2020). Haemolytic anaemia: a consequence of COVID-19. BMJ Case Rep.

[6] Quinn R, Murakhovskaya I (2021). SARS-CoV-2 and autoimmune cytopenia. Hemato.

[7] Brito S, Ferreira N, Mateus S, Bernardo M, Pinto B, Lourenço A, Grenho F (2021). A case of autoimmune hemolytic anemia following COVID-19 messenger ribonucleic acid vaccination. Cureus.

[8] Hindilerden F, Yonal-Hindilerden I, Akar E, Yesilbag Z, Kart-Yasar K (2020). Severe autoimmune hemolytic anemia in COVID-19 i̇nfection, safely treated with steroids. Mediterr J Hematol Infect Dis.

